# Three new PAX6 mutations including one causing an unusual ophthalmic phenotype associated with neurodevelopmental abnormalities

**Published:** 2007-04-02

**Authors:** Anouk Dansault, Gabriel David, Claire Schwartz, Carolina Jaliffa, Véronique Vieira, Guillaume de la Houssaye, Karine Bigot, Françise Catin, Laurent Tattu, Catherine Chopin, Philippe Halimi, Olivier Roche, Nicole Van Regemorter, Francis Munier, Daniel Schorderet, Jean-Louis Dufier, Cécile Marsac, Daniel Ricquier, Maurice Menasche, Alfred Penfornis, Marc Abitbol

**Affiliations:** 1 EA n 2502 du Ministère de la Recherche, de l'Enseignement Supérieur et la Technologie, Center de Recherches Thérapeutiques en Ophtalmologie (CERTO), Université René Descartes-Paris V, Faculté de Médecine René Descartes-Site Necker, Paris, France; 2 CNRS UPR 9078, Faculté de Médecine René Descartes-Site Necker, Paris, France; 3 Service d'Ophtalmologie du CHU de Besançon, Doubs, France; 4 Service de Neuro-Imagerie du CHU de Besançon, Doubs, France; 5 Service de Neurologie du CHU de Besançon, Doubs, France; 6 Centre Hospitalier Louis Pasteur de Dole, Jura, France; 7 Service d'Imagerie de l'Hopital Européen Georges Pompidou, Paris, France; 8 Service d'Ophtalmologie, Hôpital Necker-Enfants Malades, Paris, France; 9 Center de Génétique ULB, Bat. Transfusion 02, Hôpital Erasme, Buxelles, Belgique; 10 Jules Gonin Eye Hospital, Lausanne, Switzerland; 11 IRO-Institut de Recherche en Ophtalmologie, Sion, Switzerland; 12 Service d'Endocrinologie et Diabétologie du CHU de Besançon, Doubs, France

## Abstract

**Purpose:**

The *PAX6* gene was first described as a candidate for human aniridia. However, *PAX6* expression is not restricted to the eye and it appears to be crucial for brain development. We studied *PAX6* mutations in a large spectrum of patients who presented with aniridia phenotypes, Peters' anomaly, and anterior segment malformations associated or not with neurological anomalies.

**Methods:**

Patients and related families were ophthalmologically phenotyped, and in some cases neurologically and endocrinologically examined. We screened the *PAX6* gene by direct sequencing in three groups of patients: those affected by aniridia; those with diverse ocular manifestations; and those with Peters' anomaly. Two mutations were investigated by generating crystallographic representations of the amino acid changes.

**Results:**

Three novel heterozygous mutations affecting three unrelated families were identified: the g.572T>C nucleotide change, located in exon 5, and corresponding to the Leucine 46 Proline amino-acid mutation (L46P); the g.655A>G nucleotide change, located in exon 6, and corresponding to the Serine 74 Glycine amino-acid mutation (S74G); and the nucleotide deletion 579delG del, located in exon 6, which induces a frameshift mutation leading to a stop codon (V48fsX53). The L46P mutation was identified in affected patients presenting bilateral microphthalmia, cataracts, and nystagmus. The S74G mutation was found in a large family that had congenital ocular abnormalities, diverse neurological manifestations, and variable cognitive impairments. The 579delG deletion (V48fsX53) caused in the affected members of the same family bilateral aniridia associated with congenital cataract, foveal hypolasia, and nystagmus. We also detected a novel intronic nucleotide change, IVS2+9G>A (very likely a mutation) in an apparently isolated patient affected by a complex ocular phenotype, characterized primarily by a bilateral microphthalmia. Whether this nucleotide change is indeed pathogenic remains to be demonstrated. Two previously known heterozygous mutations of the *PAX6* gene sequence were also detected in patients affected by aniridia: a de novo previously known nucleotide change, g.972C>T (Q179X), in exon 8, leading to a stop codon and a heterozygous g.555C>A (C40X) recurrent nonsense mutation in exon 5. No mutations were found in patients with Peters' anomaly.

**Conclusions:**

We identified three mutations associated with aniridia phenotypes (Q179X, C40X, and V48fsX53). The three other mutations reported here cause non-aniridia ocular phenotypes associated in some cases with neurological anomalies. The IVS2+9G>A nucleotide change was detected in a patient with a microphthalmia phenotype. The L46P mutation was detected in a family with microphthalmia, cataract, and nystagmus. This mutation is located in the DNA-binding paired-domain and the crystallographic representations of this mutation show that this mutation may affect the helix-turn-helix motif, and as a consequence the DNA-binding properties of the resulting mutated protein. Ser74 is located in the PAX6 PD linker region, essential for DNA recognition and DNA binding, and the side chain of the Ser74 contributes to DNA recognition by the linker domain through direct contacts. Crystallographic representations show that the S74G mutation results in no side chain and therefore perturbs the DNA-binding properties of PAX6. This study highlights the severity and diversity of the consequences of *PAX6* mutations that appeared to result from the complexity of the *PAX6* gene structure, and the numerous possibilities for DNA binding. This study emphasizes the fact that neurodevelopmental abnormalities may be caused by *PAX6* mutations. The neuro-developmental abnormalities caused by *PAX6* mutations are probably still overlooked in the current clinical examinations performed throughout the world in patients affected by *PAX6* mutations.

## Introduction

The *PAX6* gene was first described in 1991 as a candidate for human aniridia [1]. This congenital abnormality is a bilateral panocular disorder affecting 1 in 64,000 to 96,000 in the general population. It is characterized by the absence of an iris and is associated with other eye abnormalities including cataract, optic nerve malformations, coloboma, and foveal hypoplasia (OMIM 106210). These defects are shared with the small eye (*Pax6*+/-) mouse, a model of the human syndromes. *PAX6* is also involved in other anterior segment malformations in addition to aniridia and associated anomalies including Peters' anomaly [2]. Embryonic development of the eye is affected in cases of Peters' anomaly, leading to corneal clouding and variable iridolenticulocorneal adhesions that may occur as an isolated ocular abnormality or together with other ocular defects.

**Figure 1:**
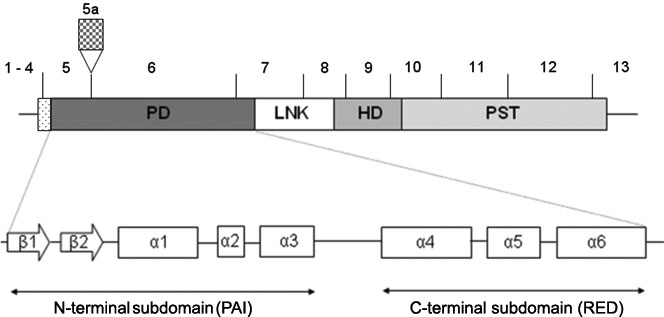
Structure of the *PAX6* gene. *PAX6* contains three highly conserved domains: a 128 amino acid DNA-binding paired domain (PD), a 60 amino acid DNA-binding homeodomain (HD), which binds different DNA target sequences, and a Proline-Serine-Threonine (PST)-rich transactivation domain.

The *PAX6* gene (NC_000011) is located on chromosome 11p13, and homologues have been found in diverse species. The gene contains 14 exons, one of which is alternatively spliced. Pax6 protein is a transcription factor containing three highly conserved domains (Figure 1), and there are two major isoforms: Pax6(-5a), comprising 422 amino acids, and PAX6(+5a), comprising 436 amino acids resulting from the insertion of a 14 amino acid-long sequence in the paired domain (PD). This insertion abolishes Pax6 DNA-binding properties [3] and seems to induce, when the Pax6(+5a) isoform is overexpressed, a developmental cascade modifying the neuronal architecture of the retina [4].

The PD comprises two separate helix-turn-helix motifs that form two subdomains (Figure 1): a NH_2_-terminal subdomain (NTS) and a COOH-terminal subdomain (CTS) that act on different target sequences [3]. The PD and the binding homeodomain (HD) recognize different DNA-binding sequences, and thus Pax6 has the potential to recognize diverse DNA-binding sites in a large variety of target genes.

PAX6 is expressed in the developing iris, lens, ciliary body, corneal epithelium, and retina. Upon completion of eye development, PAX6 expression remains in the neural retina, the lens epithelium, and the cornea. Furthermore, in combination with additional transcription factors, the level and spatial distribution of PAX6 expression in the developing eye are important for the establishment of the eye axes. For example, PAX2-PAX6 is critical in defining the optic stalk/optic cup boundary, while PAX6-cVAX and TBX5 expression mediates dorsoventral patterning of the eye [5]. A dose-dependent effect of the *PAX6* gene product has been described and the range of eye abnormalities seems to correlate with the level of *PAX6* activity [6]. However, *PAX6* expression is not restricted to the eye. It is the only PAX gene expressed in the telencephalon, the diencephalon, in the caudal part of the rhombencephalon, in the myelencephalon and in the spinal cord. It appears to be crucial for brain development [7]. However, it is not expressed in the mesencephalon [8]. The *PAX6* gene is also expressed in the postnatal and adult brains [9] and in the pancreas [10].

The expression pattern of the *PAX6* gene is consistent with both the combination of diverse ocular phenotypes observed and the distribution of neurological manifestations in isolated patients (sporadic cases) and in pedigrees (familial cases) with *PAX6* mutations. Impairment of PAX6 activity will obviously affect the expression patterns of its target genes, and there is substantial evidence of associations between *PAX6* mutations, brain development abnormalities, cognition disorders [11], as well as pancreatic endocrine disorders [12].

We screened a large spectrum of patients who presented with aniridia phenotypes, Peters' anomaly, and other anterior segment malformations. We report three new mutations and two known mutation of the *PAX6* nucleotide sequence. We describe in detail one familial mutation of the *PAX6* gene associated with a complex phenotype involving ocular malformations and neurodevelopmental anomalies. We also report an intronic nucleotide change, IVS2+9G>A, in a patient with a microphthalmia phenotype. In addition, we report two previously identified nonsense mutations, Q179X and C40X.

## Methods

### Patients

All participants gave their informed consent according to the Bioethics Laws of the European Union and France, and according to the Helsinki Declaration. The families were recruited in the "Département d'Ophtalmologie, Centre Hospitalier Necker-Enfants Malades" (France), in the "Institut de Recherches en Ophtalmologie" (Switzerland) and in "Hôpital Erasme" (Belgium). We screened the *PAX6* gene in 78 persons, from 0 to 73 years (affected patients and in most instances their relatives), including unrelated families and apparently isolated cases. The patients were classified into three groups. Group 1 was composed of 36 patients (22 isolated patients and 14 affected members belonging to three unrelated families) who were affected by aniridia associated or not with other abnormalities. Group 2 contained 10 patients with Peters' anomaly. Group 3 comprised 32 patients (13 apparently isolated patients and 19 affected members belonging to two different families), who were suffering neither aniridia nor Peters' anomaly but were affected by diverse ocular and, in some cases, neurological anomalies. We also established a control group of 200 unrelated normal individuals devoid of any eye disease, who were screened for *PAX6* nucleotide changes. Table 1 gives the ages of the affected patients at the time of phenotyping.

**Table 1:** Ages of affected patients.

Isolated Case A														
Age (year)	1													
Family B														
Patients	B-I-2	B-II-1												
Age (year)	45	21												
Family C														
patients	C-I-2	C-II-1												
Age(years)	20	1												
Isolated case D														
Age (year)	9													
Family E														
Patients	E-I-1	E-II-1	E-II-2											
aqe (year)	42	19	18											
Family F														
Patients	F-l-2	F-ll-2	F-lll-4	F-IV-2	F-IV-5	F-IV-8	F-V-1	F-V-3	F-V-4	F-V-5	F-V-6	F-V-7	F-V-11	F-V-14
aqe (year)	-	-	20	3	I	at birth	1	1	1	3	at birth	at birth	1	at birth
Family F*														
patients	F-I-2	F-II-2	F-III-4	F-IV-2	F-IV-5	F-IV-8	F-V-1	F-V-3	F-V-4	F-V-5	F-V-6	F-V-7	F-V-11	F-V-14
aqe (year)	-	-	73	51	49	31	26	21	13	10	26	25	4	at birth

We indicate in this table the ages of patients at the time of their first clinical phenotyping, in the University Hospital of Lieges, Belgium, in the Jules Gonin Hospital of Lausanne, Switzerland as well as in the Necker-Enfants Malades Hospital of Paris (Case A, Family B, Family C, Case D, and Family E). All Family F members were examined in the Hospitals of Dôle, Besançon, and Dijon. Two patients from Family F were initially examined in the Necker-Enfants Malades Hospital of Paris. Asterisk indicates the ages of affected patients during their phenotyping, which was performed in November 2003 at the Hospitals of Dôle, Besançon, and Dijon within the framework of this study. For patients F-I-2 and F-II-2, now deceased, the ages of the diagnosis were not available (dashes).

All the patients included in this report submitted to a thorough ophthalmic examination. Some patients also underwent a neurological examination, brain magnetic resonance imaging (MRI) and a complete systematic neurological and endocrinological evaluation (tests for follicle-stimulating hormone [FSH], luteinizing hormone [LH], adreno-corticotrophic-hormone [ACTH], prolactin, growth hormone, somatomedin C, thyroid-stimulating hormone [TSH], cortisol, estradiol concentrations, synacthene, and luteinizing hormone-releasing hormone [LHRH]).

### Intelligence Quotient testing

The Intelligence Quotient values of all the affected and nonaffected members of the large family F were measured using the Stanford-Binet IQ test version 5.

### DNA amplification and sequencing

DNA was extracted from blood lymphocytes by a proteinase K digestion technique. Each exon of the *PAX6* gene was amplified from genomic DNA by polymerase chain reaction (PCR) using primers chosen by ourselves and those described by Love et al. [13] and detailed in Table 2. PCR used 60 ng of each patient's genomic DNA as a template, 20 pmol of each primer, 10 mM Tris-HCl at pH 8.3, a MgCl_2_ concentration depending upon the exon amplified, 50 mM KCl, 1.5 units of *Thermophilous aquaticus* (Taq) DNA polymerase, and dNTP. PCR consisted of 30 cycles and was carried out in an automated MJC thermal cycler (PTC 225). All fragments were purified using the Concert kit using NucleoFast 96 PCR plates and then analyzed by direct sequencing using an ABI PRISM 3100 DNA sequencer.

**Table 2:** List of primers used and the PCR product size.

Exon	Primer	Sequences (5'-3')	Amplicon
1	1F	AGGGAACCGTGGCTCGGC	207
	1R	GGGTGAGGGAAGTGGCTGC	
2	2.1F	TTATCTCTCACTCTCCAGCC	178
	2.1R	CTGTTGTTGCTTGAAGACCAC	
	2.2F	AAACTCTCACCAGCAACTCC	197
	2.2R	GGAGACCTGTCTGAATATTGC	
3	3F	GGACGTATGCTGTTGAACCAC	166
	3R	TGAGCCCAAAGCAGCCACCA	
4	4F	TGCAGCTGCCCGAGGATTAA	133
	4R	CCGAAGTCCCAGAAAGACCA	
5	5.1F	CTCTTCTTCCTCTTCACTCTG	163
	5.1R	CGCTGTGAGCTAGCTCTAC	
	5.2F	CGGTGGTGTCTTTGTCAACG	170
	5.2R	AGAGGGCGTTGAGAGTGG	
5a	5aF	CTCTACAGTAAGTTCTCATACC	173
	5aR	GGAAGTGGACAGAAAACCAC	
6	6.1F	TGGTTTTCTGTCCACTTCCC	167
	6.1R	GCACTCCCGCTTATACTGG	
	6.2F	CCGAGAGTAGCGACTCCAG	184
	6.2R	AGGAGAGAGCATTGGGCTTA	
7	7.1F	GTGAGCTGAGATGGGTGA	175
	7.1R	CTTCCGGTCTGCCCGTTC	
	7.2F	ATGGGCGCAGACGGCATG	171
	7.2R	GACAGGCAAAGGGATGCAC	
8	8.1F	CCCTTTTGGAGGCTCCAAG	185
	8.1R	GATGTTCTATTTCTTTGCAGC	
	8.2F	TCCAACGGAGAAGATTCAG	176
	8.2R	TCTTTGTACTGAAGATGTGGC	
9	9F	GGAGGTGGGAACCAGTTTGA	195
	9R	GTGAAACTGCACAGTCTCTC	
10	10.1F	CTCGACGTAGACACAGTGC	175
	10.1R	AATTGGTTGGTAGACACTGG	
	10.2F	TCAGAGAAGACAGGCCAGC	153
	10.2R	CCCGGAGCAAACAGGTTTAA	
11	11F	GGGCTCTGACTCTCACTCTG	191
	11R	TTATGCAGGCCACCACCAGC	
12	12.1F	GCTGTGTGATGTGTTCCTCA	168
	12.1R	GACTGTTCATGTGTGTCTGC	
	12.2F	CTGCATGCTGCCCACCAG	163
	12.2R	AAGAGAGATCGCCTCTGTGC	
13	13F	CATGTCTGTTTCTCAAAGGG	202
	13R	CCATAGTCACTGACTGAATTAACAC	

For each pair of primers, the amplicon size is indicated in base pairs. F: forward primer; R: reverse primer. Exons 2, 5, 6, 7, 8, 10, and 12 were amplified in two overlapping fragments. In each case, primer pair (.1F and .1R) amplifies the most upstream 5' part of the target genomic DNA, and primer pair (.2F and .2R) amplifies the most downstream 3' part of the target genomic DNA encompassing the specific exonic sequences. Sequences are given in the 5'-3' direction.

### Crystallographic views

We analyzed the crystallographic structures of some of the mutant proteins to visualize the consequences of the amino acid changes (missense mutations). All crystallographic views were obtained and derived from the structural model determined from X-ray diffraction (PDB id:6PAX). The structures of the mutated residues were generated using SwissProt PDB viewer and viewed in Rasmol or PyMol.

## Results

In Group 1, which contained patients affected by aniridia, we found a novel mutation and two previously described *PAX6* mutations. In Group 3, composed of patients with diverse ocular manifestations and some associated neurologic abnormalities, we found two novel mutations and an intronic nucleotide change. However, we did not find any *PAX6* mutations in Group 2, which was composed of patients with Peters' anomaly. All identified *PAX6* mutations are summarized in Table 3.

**Table 3:** Summary of detected mutations and changes.

patients	patient group	Nucleotide change		Amino acid change		Exon
		PAX6(-5a)	PAX6(+5a)	PAX6(-5a)	PAX6(+5a)	
A	1	972C>T	1014C>T	Q179X	Q193X	8
B-I-2, B-II-1 and B-II-2	1	555C>A	555C>A	C40X	C40X	5
C-I-2 and C-II-1	1	579delG	621delG	V48fsX53	V62fsX67	6
D	3	IVS2+9G>A	IVS2+9G>A			
E-I-1, E-II-1 and E-II-2	3	572T>C	572T>C	L46P	L46P	5
F-I-2, F-II-2, F-II-4,						
F-IV-2, F-IV-5, F-IV-8, F-V-1, F-V-3, F-V-4, F-V-5, F-V-6, F-V-7, F-V-11, F-V-14	3	655A>G	697A>G	S74G	S88G	6

**Figure 2:**
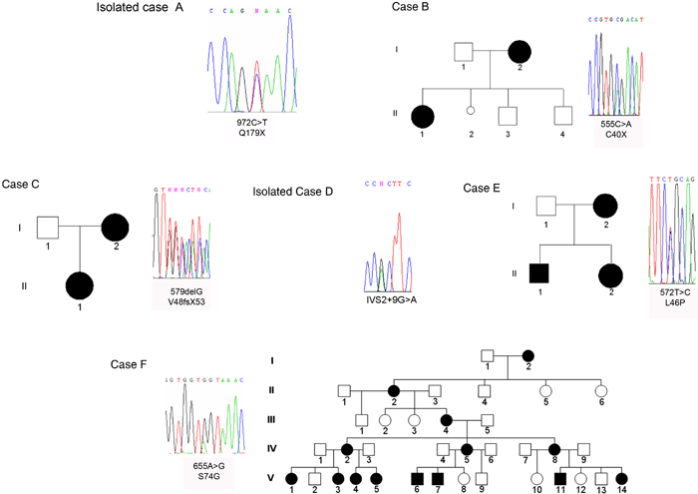
Pedigrees and mutation sequences in the *PAX6* gene. Males and females are represented by squares and circles, respectively, and affected family members are darkened symbols. Partial DNA sequence chromatographs of *PAX6* genomic sequences from affected individuals in two isolated cases (A and D) and in four unrelated families (B, C, E and F) are shown. The accurate mutations in families and isolated patients were indicated as closely as possible to each sequence and their names were provided according to the international nomenclature for human gene mutations. The nucleotide numbering of the mutations was based on the sequence of the PAX6(-5a) isoform.

We show the pedigrees of the affected patients whenever they were available with certainty. We also indicate the sequence of the six mutations reported in this study. Each mutation is associated with the affected patients from the clinical group 1 corresponding to aniridia phenotypes or from the group 3 corresponding to other ocular phenotypes associated in one large family (F) with neurological anomalies. A and D are isolated cases whereas B, C, E, and F refer to familial cases (see Figure 2). The exon in which the mutated amino acid residue is located is mentioned. Each mutation is named according to the nucleotide change and the corresponding amino acid modification for the two major isoforms: Pax6(-5a), comprising 422 amino acids, and PAX6(+5a), comprising 436 amino acids resulting from alternative splicing.

### Mutations found in Group 1

We found a 972C>T (Q179X) transition (Figure 2) in isolated case A, who had presented with bilateral aniridia and no other associated abnormality.

Family B consisted of six members of which one was a stillborn child (II-2; Figure 2). We identified a 555C>A (C40X) mutation in exon 5 in two affected patients, I-2 and II-1. Both presented with bilateral aniridia associated with bilateral cataracts, nonvascularized corneal opacities, and bilateral glaucoma that was more severe in patient II-1 than in patient I-2. Both underwent endocrinological examinations that revealed a low ACTH concentration exclusively in patient II-1.

Family C consisted of three members (Figure 2). Affected members I-2 and II-1 carried a heterozygous 579delG (V48fsX53) mutation in exon 6. Both of the affected members presented with bilateral aniridia associated with bilateral congenital cataract, bilateral foveal hypolasia, and nystagmus.

### Mutations found in Group 3

In isolated case D, we identified a heterozygous IVS2+9G>A nucleotide change (Figure 2). This patient presented with a microphthalmia phenotype associated with bilateral congenital microcornea, powdered cataract, nystagmus, and partial sclerocornea, as well as a band-shaped keratitis in the left eye. The examination of this patient under general anesthesia, when he was one year old, allowed us to estimate the axial length of his right eye at a value of 12 mm and the axial length of his left eye at a value of 13 mm. The visual electrophysiological investigations (electro-oculogram, electro-retinogram and visual-evoked potentials) and the brain computed tomography scan were surprisingly normal.

**Figure 3:**
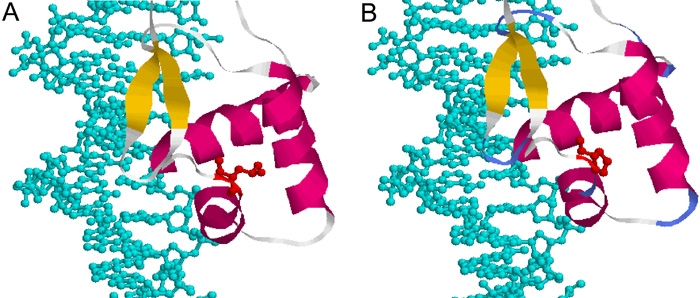
Crystallographic views of leucine 46 to proline mutation found in family E. Represented are the interactions between DNA (blue) and the helix-turn-helix motif affected by the amino acid change (red) in the wild-type protein (**A**) and in the mutated protein (**B**). The mutated Leu 46 is located just after the second helix in the NH_2_-terminal subdomain of the PAX6 paired domain (PD). The substitution of a proline for a leucine modifies the structure of the second helix and affects the HTH motif conformation. This could alter the position of the third helix, which recognizes the DNA. The readers should be aware that we standardized the numbering of the amino acid residues of the paired domain. We allocated a number to each amino acid residue that corresponded to its location vis-a-vis the first methionine, initiating the PAX6 short protein isoform (-5a). However, the X-ray crystallography specialists use to allocate a numbering to the amino acids of the PAX6 PD which starts at the first amino acid of this PD.

Family E consisted of four members. We found an 572T>C (L46P) mutation in exon 5 of affected patients I-1, II-1 and II-2 (Figure 2). These patients had presented with bilateral microphthalmia associated with bilateral congenital cataract, bilateral glaucoma. and nystagmus. All affected patients of this family had an abnormally shaped and shortened anterior chamber and were at risk of closed-angle glaucoma. In addition, they had a strong myopia (greater than or equal to 5 diopters). The axial measurements of the distance between the posterior face of the lens and the retina were abnormally high and always superior to 23 mm in all affected patients of this family and completely compatible with the degree of myopia detected in each patient's eye. We previously did not find any *PITX2* nor *FOXC1* mutation in these patients. We investigated consequences of this PAX6 mutation by generating crystallographic views of the amino acid change. The mutated L46 is located in the NH_2_-terminal subdomain of the PAX6 PD, just after the second helix (Figure 3A). The substitution of a leucine by a proline (Figure 3B) modifies the structure of the second helix and affects the helix-turn-helix (HTH) motif conformation.

**Figure 4:**
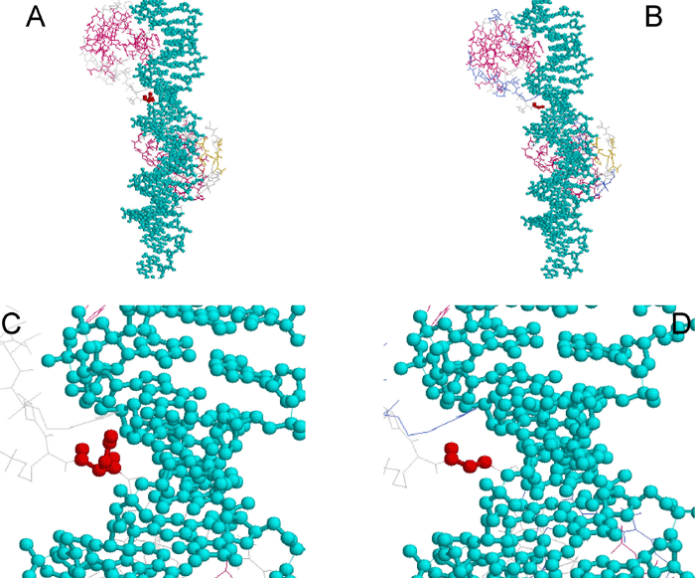
Crystallographic views of the serine 74 to glycine mutation found in family F. Represented are the interactions between the DNA (blue) and PAX6 PD red. **A** and **B** show global views while **C** and **D** represent magnified views of the linker region of the PD (residue 61-76) in which the mutated Ser74 is located. This region makes many contacts with the minor groove of the DNA strand. In the wild-type protein (**A** and **C**), the Ser-74 (red) side chain contacts the DNA. In the mutated protein (**B** and **D**), the amino acid change leads to the loss of the side chain and increases the distance between the target genomic DNA and the PAX6 linker domain, making direct contact between DNA and PAX6 impossible.

**Figure 5:**
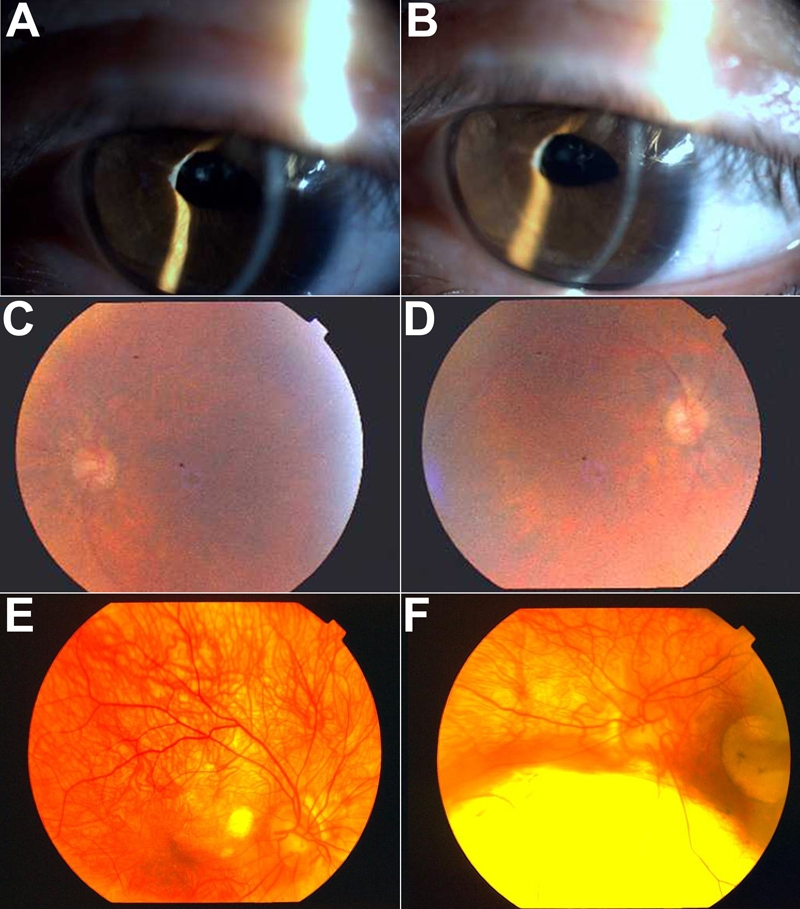
Biomicroscopic observations in affected patients from the family F who carry the PAX6 serine 74 to glycine mutation. **A** and **B** show slit lamp aspects of the ocular anterior segment as well as of the lens of patient IV-5. **C**, **D**, **E**, and **F** are funduscopy photographs of the foveal hypoplasia of patient IV-8 (**C** and **D**) and the colobomas in patient V-11 (**E** and **F**).

**Figure 6:**
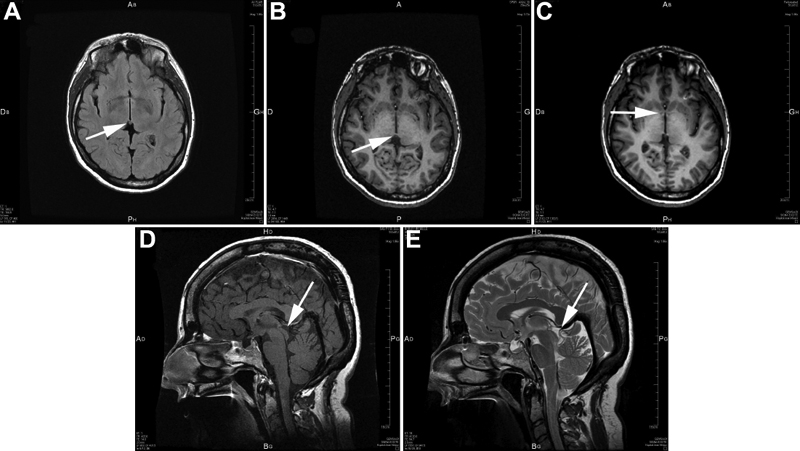
Brain MRI abnormalities in patient IV-5 from family F with the PAX6 serine 74 to glycine mutation. We show different MRI views of patient IV-5 from family F. **A**, **B**, and **C** correspond to MRI horizontal views, while **D** and **E** correspond to MRI sagittal views. MRI in **A** shows a small structure that might correspond to the habenula (arrow). MRI in **B**, **D**, and **E** reveals the absence of the pineal gland (arrow). MRI in **C** shows hypoplasia of the anterior commissure of the brain (arrow).

Family F consisted of 36 members, distributed in five generations, of whom 14 members were affected by a 655A>G (S74G) mutation in exon 6 (Figure 2). The S74G mutation resulted in no side chain, and crystallographic analysis (Figure 4) shows a distance increase between the DNA and the atoms of the PAX6 PD. Most patients presented either with minor or major bilateral foveal hypoplasia (Figure 5C and Figure 5D) or, in some rare instances, either macular coloboma or a major posterior coloboma (Figure 5E and Figure 5F). All affected members presented with congenital bilateral multidirectional nystagmus probably due to foveal or macular alterations. Moreover, they also displayed either a congenital cataract (rarely) or, more commonly, a precocious infantile progressive bilateral cataracts with substantial phenotypic variability between the affected family members (Figure 5). Some of the patients had highly unusual ophthalmic phenotypes, which in a few cases differed bilaterally. Several affected patients of this family suffered neurodevelopmental defects and/or mental retardation and/or epilepsy. At least four patients V-6, V-7, V-11, and V-14, had epileptic seizures confirmed during their sojourn in the hospital. Some affected patients presented with permanent cognitive impairments associated with neurological abnormalities, detected with certainty at birth for patients V-6 and V-7. At least since birth, patient V-7 suffered a general cortical atrophy associated with a large cyst of the septum lucidum separating the horns of the lateral ventricles. Patients IV-5 and V-7 presented with spontaneous multidirectional nystagmus aggravated by different movements of ocular globes and a moderately static cerebellar syndrome. Brain MRI imaging of patient IV-5 revealed a hypoplasia of the anterior commissure (Figure 6C) and the absence of the pineal gland (Figure 6). Patient IV-8 had signs of a mild pyramidal syndrome, whereas patient V-6 presented with unambiguous pyramidal syndrome. All patients from this family were examined by two separate neurologists in two different hospitals. All affected members of this family presented with a mild cerebellar syndrome, which in some affected patients was clinically obvious spontaneously without any additional clinical examination required for diagnosis confirmation. To this constant neurological abnormality, the severity of which could vary significantly from one affected patient to the other, were unfrequently associated neurological manifestations including epilepsy. The cognitive impairments and an abnormal control of emotivity were constantly present in all affected members of this family. The IQ testing of all affected and unaffected members of the large family F provided IQ values constantly just below the normal values in most affected members, but low IQ values in some severely affected patients. None of the unaffected patients presented any abnormal IQ value. None of the family members who did not carry the mutation displayed any neurological or cognitive impairment nor any abnormal control of humor or emotivity. Endocrinological investigations showed patient IV-5 had a low response and patient IV-8 a blunted response to the synacthen test.

## Discussion

The nucleotide mutations we report in Group 1 are associated with both sporadic (case A) and familial cases of aniridia (cases B and C). These patients have various aniridia phenotypes, with various associated ocular disorders. All these associated anomalies fall within the wide clinical spectrum of abnormalities often observed in cases of aniridia.

The first mutation, Q179X, we describe (case A) is a previously known mutation [14]. It appeared that the patient carries a de novo mutation or that one of his parents has a germinal mosaicism for this mutation. The mutation leads to a premature stop codon that truncates the paired domain close to its COOH-terminal part.

The nucleotide change identified in family B introduces a stop codon (C40X) that truncates PAX6 within the NH_2_-terminal half of the PD. This mutation is a recurrent protein truncation mutation that is always associated with aniridia, as seen in a search of the Human PAX6 Allelic Variant Database. Patient II-1 presented with a low ACTH concentration. This phenotype can be explained by the expression of the *PAX6* gene in the hypothalamus. This mutation could lead to a degradation of mRNA by nonsense-mediated decay. In this case, no truncated protein would be produced. However, some mRNA harboring premature stop codons escape the degradation pathway and make truncated protein synthesized [15]. If the altered mRNAs are not degraded by nonsense-mediated decay and are translated, the mutated protein would lack the C-terminal subdomain of the PD and the homeo- and PST-domains. We suggest that the resulting 36 amino acid polypeptide cannot bind normally to the genomic DNA target sequences. Thus, the resulting protein is unlikely to have the transactivating activities that are crucial for normal eye development.

In family C, we identified a deletion, 579delG, that creates a frameshift in the reading and introduces a stop codon in position 53 (V48fsX53). If the altered mRNAs are not degraded by nonsense-mediated decay and are translated, the mutated protein would only contain 52 amino acids of the NH_2_-terminal part of PAX6. The resulting truncated protein would lack the homeo- and PST-domains and would certainly be nonfunctional.

In many affected patients from Group 1, we did not detect any mutations in exons of the *PAX6* gene, although *PAX6* is, thus far, the only gene associated with aniridia. These results can be explained by the technique used in this study as well as by a possible overlooked clinical bias in patient recruitment for this study. Mutations may be located outside the studied genomic region, in enhancers or silencers, in the promoter region, or in distant intron sequences, for example. We can increase the detection rate using several techniques: Southern blotting, quantitative genomic PCR, illegitimate RT-PCR with total RNA extracted from lymphoblastoid cell lines or from white blood cells, and high resolution karyotyping. Furthermore, there are published cases of aniridia-like phenotypes related to unknown genes [16]. Although *PAX6* appears to be the major, and maybe even the only, gene involved in autosomal dominant aniridia, other aniridia clinical forms may be due to yet to be identified genes.

We screened ten patients with Peters' anomaly for *PAX6* mutations (Group 2). We found no *PAX6* mutation in this group of patients, whereas the PAX6 mutation database reports several additional PD missense changes with the Peters phenotype [17]. Peters' anomaly may be caused by mutations in several genes, including *PITX2* [18], *FOXC1* [19], *CYP1B1* [20], *EYA1* [21], and *FOXE3* [22]. The phenotype of these patients is therefore probably a direct consequence of mutations in one of these genes that are also involved in eye development rather than to mutations in the *PAX6* gene.

As *PAX6* is involved in the eye and the central nervous system development, we studied patients with various anterior segment abnormalities and also neurological impairments, frank neurological abnormalities as well as mental retardation (Group 3). We identified an intronic transition (IV2+9G>A) in sporadic case D belonging to Group 3. This change is observed in an isolated sporadic case and was not been found in our control group. Thus, the IV2+9G>A could likely be a mutation or a rare polymorphism. Additional experiments based on the illegitimate transcription method are required for demonstrating the pathogenicity of this nucleotide change. Illegitimate transcription relies upon the RT-PCR of total RNAs extracted from white blood cells or immortalized fibroblastic or lymphoblastic cell lines from the patient. It could allow us to detect possible alternative splicing abnormalities affecting the length and the sequence of the *PAX6* transcripts. Unfortunately, the materials necessary for performing these experiments are not available in our laboratory. The occurrence of this intronic change and the growing rate of discovery of genuine novel intronic mutations causing several inherited diseases highlight the need to study both genomic DNA and RNA. In so doing, this would increase the likelihood of identifying disease-causing mutations in intronic sequences.

**Figure 7:**
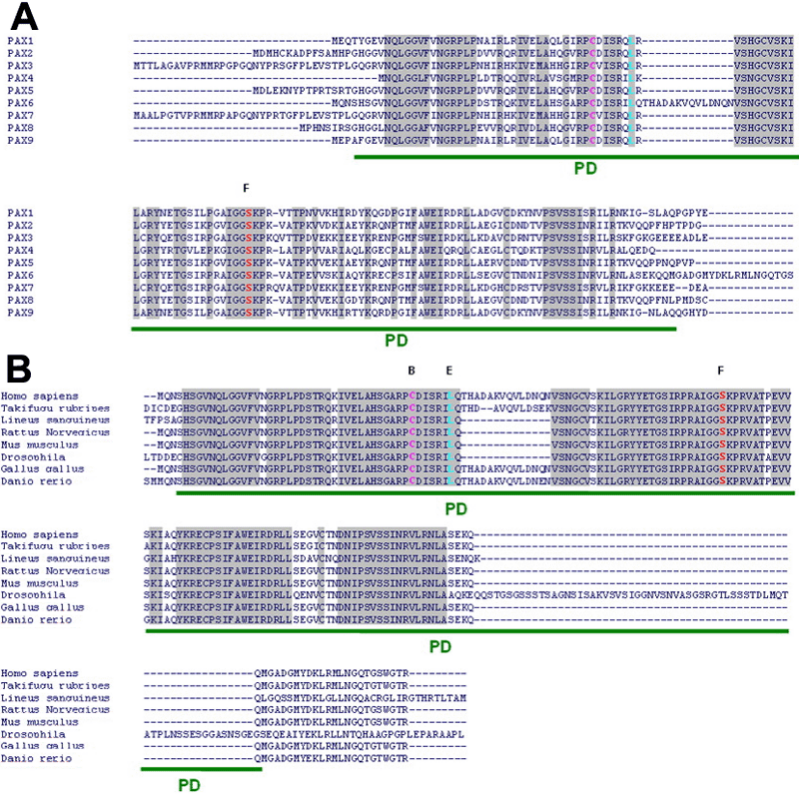
Amino acid sequence alignment of the human PAX6 paired domain. **A** shows alignment of human PAX6 protein sequence with seven other species: *Takifugu rubripes*, *Lineus sanguineus*, *Rattus norvegicus*, *Mus musculus*, *Drosophila melanogaster*, *Gallus gallus*, and *Danio rerio*. The PAX6 PD is highly conserved throughout species. The amino acids affected by mutation in the PD reported in this study are shown in color. **B** shows the PD amino acid sequence alignment of all PAX genes (PAX1-5 and PAX7-9). The amino acids affected by mutation in the PD reported in this study are shown in color. The PD is highly conserved troughout all PAX proteins.

In family E, we identified a L46P mutation. The residue concerned is in the NH_2_-terminal subdomain of the PAX6 PD (residues 4-63) immediately downstream from the second helix, and is highly conserved between PDs (Figure 7). This domain has an HTH motif. The third helix recognizes the DNA binding proteins [23,24]. The L46P mutation is at the end of the second helix and specifically modifies its structure due to the proline being in either cis or trans conformation. This mutation affects the HTH motif. This could modify the position of the third helix, which recognizes the DNA. Figure 3 shows where the amino acid is located (at the end of the second helix) and is based on the structure obtained by X-ray diffraction (PDB id:6PAX). The Leu 46 residue does not appear to directly contact the DNA and thus the DNA-binding activity of the COOH-terminal subdomain of the PD carrying a mutated leucine at position 46 may be conserved, although the mutated protein does not express strong transactivation activity. Chao et al. [25] described a mutation that affects the same leucine residue (L46R): Two members of a family carried the same mutation but had different phenotypes, and none of them had aniridia. This phenotype appears somewhat similar to the phenotype of our patients, in which the same amino acid residue is mutated, albeit not to the same amino acid. Chao et al. [25] found that the mutated PAX6 protein did not bind DNA and was less able than the wild-type PAX6 to transactivate genes. These results suggest that similar phenotypes, and in particular phenotypes without aniridia, may be related to mutations affecting the same amino acids of the *PAX6* gene, the major cause of aniridia.

We identified a S74G missense mutation in exon 6 in all affected members of family F. This residue is in the PAX6 PD linker region (residues 64 to 79) connecting the NTS and the CTS. This linker is highly conserved in all known PD proteins (Figure 7), and it makes numerous contacts with the minor groove of the DNA strand (Figure 4A,B); it is essential for DNA recognition and DNA binding. Ser74 directly contacts the adenine at position 14, in the oligonucleotide sequence including the PAX6 binding site, used by Xu et al. [23,24] for cocrystallization to study the interactions between the PAX6 paired domain and its core target genomic DNA binding sequence. This contact is made with no water molecule found between Ser74 and adenine 14 (Figure 4C,D). Ser74 is essential for DNA binding, because its side chain contributes to DNA recognition by the linker domain through direct contacts. The side chain connects with N-3 of adenine 14 and also with the phosphate group of guanine 15. Any mutation that leads to a residue without a side chain or with a differently charged side chain will lead to incorrect DNA binding in this linker region. The S74G mutation results in no side chain and thus perturbs the DNA-binding properties of PAX6. In silico, crystallographic analysis shows that with this mutation, the distance between the DNA and the atoms of the PAX6 linker domain are greater than 5Å, which is twice the distance observed for the wild-type, and thus the direct contacts of the PD with the DNA (adenine 14 or guanine 15) are lost. These abnormally large distances between atoms of the target DNA and atoms of the PAX6 PD linker domain may well be the structural basis of the abnormal DNA-binding properties of PAX6. Additional biochemical experiments are required for demonstrating unambiguously the pathogenicity of this mutation at the atomic level and for confirming or invalidating the pathogenic model supported by our modeling. The major ophthalmic manifestations caused by the S74G mutation in all affected members of family F are bilateral foveal hypoplasia and bilateral congenital cataract. This unusual ocular phenotype caused by the S74G mutation in *PAX6* gene is similar to that described by Hanson et al. [26] and is associated with a G64V substitution affecting a specific glycine amino-acid residue located just beyond the third alpha-helix of the NH_2_-terminal paired subdomain. Glycine is completely invariant at this position in all paired domain proteins characterized (Figure 7). This G64V mutation was described in a family in which the mother and her two children appeared to have an ocular syndrome involving presenile cataract and foveal hypoplasia. The mother had congenital nystagmus with bilateral cataracts in addition to bilateral foveal hypoplasia and abnormalities of the peripheral corneal epithelium. The son had bilateral nystagmus from early infancy and bilateral mild lens opacities later in life. The daughter also had bilateral nystagmus, congenital bilateral cataracts, as well as tilted optic discs and bilateral foveal hypoplasia [26]. This report [26] and our observation of bilateral cataracts in all affected members of family F highlight the importance of both residues Gly64 and Ser74 in the transactivation properties of the paired domain vis a vis genes involved in lens development and maintenance of its transparency. Indeed, *PAX6* has fundamental roles in the developing lens since the earliest stages of its formation as demonstrated by a recent elegant work showing the crucial importance of PAX6 interactions with transcription factors such as SIX3 and SOX2 in the induction of the lens placode [27]. Moreover, other genes encoding crystallins are transcriptionally controlled by PAX6: αA-crystallin, αB-crystallin, γF-crystallin, β-crystallin, and ε-crystallin [28].

The S74G mutation is particularly interesting because it is associated with unusual ocular phenotypes and, more important, with a constant cerebellar syndrome in all affected patients of one large family. Most affected patients of this family displayed nearly constantly cognitive impairments with a low IQ. All unaffected patients of family F have a normal IQ. Some patients of this family had epilepsy, while others displayed variable neurological deficits along with severe cognitive deficiencies. At least one patient of this large family (V-7) was affected by a severe form of mental retardation without any karyotype abnormality. These clinical manifestations caused by a mutation in the PAX6 PD highlight the early and extensive role of PAX6 in eye and central nervous system development and its persistent contribution to adult human brain function. Missense mutations of the PD, such as S74G, considerably impair the interaction of the PD with its DNA target sequences.

Given the *PAX6* gene expression pattern both in mouse and human during development and adulthood, it is not surprising that such mutations can trigger neurodevelopmental defects associated with permanent neurological deficiencies in adults, including anterior commissure hypoplasia, absent pineal gland (IV-5), static cerebellar syndrome (IV-8), and moderate to severe pyramidal syndrome as observed in our patients (IV-8). Patient IV-5 presented with hypoplasia of the anterior commissure. We must strongly emphasize that all affected patients carrying the S74G mutation in the large family studied and described partially in this report present with a constant cerebellar syndrome of variable severity. The variability of the phenotype observed in this family, including the variability of the neurological and cognitive deficits or impairments is consistent with the autosomal dominant mode of inheritance of this mutation. Variable expressivity and incomplete penetrance are two well known features of this mode of inheritance. These features can be explained by genetic epistratic effects or by other mechanisms beyond the scope of this report. Furthermore, PAX6 rarely acts alone on any given target gene or network of target genes. Instead, it acts with other DNA-binding proteins in complexes involving proteins belonging to diverse families of transcription factors as well as cotranscription factors such as other members of the PAX family e.g., PAX2, members of the SIX family such as SIX3, members of the dachsund0 family, the EYE ABSENT family, the SOX family, and other families of transcription factors.

Recent studies using MRI, moreover functional MRI, have shown that individuals with aniridia and cognition deficits due to a heterozygous mutation in *PAX6* have structural abnormalities of the grey matter as well as white matter deficits in the corpus callosum [11]. A subset of patients affected by *PAX6* mutations and presenting with agenesis of the anterior commissure was shown to have mild cognitive impairments [29]. The most severely affected patient of this large family, V-7 presented with a dramatic form of mental retardation associated with neurological manifestations. Sisodiya et al. [30] reported the occurrence of an absence or hypoplasia of the anterior commissure and reduced olfaction in many aniridia cases without callosal agenesis. They also suggested that *PAX6* haploinsufficiency caused more widespread neurodevelopmental anomalies in humans than first anticipated, based on the ocular and neurological observations reported in aniridia patients. Patient V-7 and his mother IV-5 had no pineal gland. One MRI study [31] showed the absence or hypoplasia of the pineal gland and absence or hypoplasia of the anterior commissure are common in patients who have aniridia and known *PAX6* mutations with diverse ocular abnormalities. This study described more accurately the consequences of *PAX6* mutations, including PD missense mutations that are usually underrepresented in aniridia. Some of the patients (V-6, V-7, V-11, and V-14) suffered from epilepsy. Epilepsy has also been reported in a study of patients affected by all types of *PAX6* mutations [31]. The affected patient IV-8 presented with an obvious static cerebellar syndrome. This observation is consistent with data collected in studies of the Sey (Small eye) mouse, showing that Pax6 is produced in cerebellar granule cells and that PAX6 influences the morphogenesis of the whole cerebellum. Mutations in the *PAX6* gene significantly affect cerebellar development [32]. Our results further support these data and highlight the role of *PAX6* mutations in structural brain anomalies and cognitive anomalies. They also allow these anatomical abnormalities to be linked to the known roles of murine Pax6 in regional differentiation, axonal guidance, and other aspects of early forebrain development [11]. Pax6 seems to play a crucial role in the developing central nervous system by transcriptional regulation of various target genes such as cell adhesion molecules (CAMs), in particular NCAM, L1CAM R-cadherin, Cadherin 6, and Cadherin 8. Studies have shown that, in *PAX6* mutants, the expression of these molecules is altered and mutations in these target genes are associated with severe brain abnormalities [28]. All these data explain how mutations in the *PAX6* gene can, directly or not, lead to cerebral impairments or frank abnormalities. In the case of the family reported here, the members who did not carry the mutation did not have these impairments. Moreover, the neurological manifestations we describe have been previously associated in the literature with *PAX6* gene mutations. Although these neurological phenotypes might be caused or influenced, in terms of severity, by nucleotide variants of other developmental genes coding proteins interacting or not with *PAX6*, we suggest that the S74G PAX6 mutation might play a causative role, in isolation or not, in the complete phenotypes reported in this family. We also observed abnormally lowered or blunted plasma cortisol response to the synacthen test in two adult patients carrying the S74G PAX6 mutation. The *PAX6* master gene is early expressed in the anterior neural plate from which the hypothalamopituitary axis and the retina are derived.

In this study, we report three novel *PAX6* mutations: two previously described mutations and a novel intronic *PAX6* nucleotide change of which the pathogenicity is not yet proved but is likely. We observed in this study that all aniridia phenotypes corresponded to nonsense mutations or deletions that led to a premature termination codon, whereas nonaniridia phenotypes were associated with missense mutations. These results are in full agreement with the genotype-phenotype correlations reported by Tzoulaki et al. [33]. They examined the Human PAX6 Allelic Variant Database and found that three-quarters of aniridia cases were caused by mutations that introduced premature termination codons into the *PAX6* open reading frame. Our mutational screening led us to identify four familial mutations corresponding to two novel transitions: one previously reported transversion and one base pair deletion. We also detected nucleotide changes in sporadic cases, one of which is a previously reported mutation corresponding to a transition and the other is a novel intronic nucleotide change, the pathogenicity of which remains to be confirmed. Three of the mutations were located in the PD (cases A, E, and F), which, as shown by Prosser and van Heyningen [34], has a higher mutation rate than the rest of the *PAX6* gene. Two of these mutations, found in cases A and E, were located in the NTS, whereas the third mutation, found in case F, was in the PD linker. There is evidence that the different DNA binding domains of PAX6 have different targets, but it has also been shown that the PD modulates the HD DNA-binding activity [35]. The two PD subdomains seemed to influence differently the DNA binding of the HD of PAX6. Singh et al. [35] analyzed the effects of two missense mutations in the PD, each one being located in one of the two subdomains. The mutation in the NH_2_-terminal subdomain resulted in a loss of function through the PD, but enhanced function mediated through the HD. The mutation in the COOH-terminal subdomain resulted in a total loss of function of the protein. Thus the severity and diversity of the consequences of *PAX6* mutations appeared to result from the complexity of the *PAX6* gene structure, and the numerous possibilities for DNA binding were due to the large variety of specific gene targets. It is our opinion that neurodevelopmental abnormalities induced by *PAX6* mutations have been underestimated for a long time. These abnormalities should systematically be investigated both by clinical examination and MRI imaging in every patient affected by aniridia or any congenital eye abnormality.
